# Treatment Outcomes and Prognostic Factors in Children with Myeloid Leukemia Associated with Down Syndrome: A Nationwide Polish Retrospective Study

**DOI:** 10.3390/jcm15197548

**Published:** 2026-09-28

**Authors:** Katarzyna Pawińska-Wąsikowska, Małgorzata Czogała, Karolina Bukowska-Strakova, Teofila Książek, Agnieszka Pac, Magdalena Samborska, Jacek Wachowiak, Anna Tomoń, Radosław Chaber, Karolina Zielezińska, Tomasz Urasiński, Anna Rodziewicz-Konarska, Krzysztof Kałwak, Marta Kozłowska, Ninela Irga-Jaworska, Bartosz Chyżyński, Paweł Łaguna, Katarzyna Muszyńska-Rosłan, Paulina Deleszkiewicz, Joanna Zawitkowska, Monika Lejman, Katarzyna Bobeff, Wojciech Młynarski, Katarzyna Mycko, Wanda Badowska, Robert Dębski, Jan Styczyński, Weronika Stolpa, Agnieszka Mizia-Malarz, Walentyna Balwierz, Szymon Skoczeń

**Affiliations:** 1 Department of Pediatric Oncology and Hematology, Institute of Pediatrics, Jagiellonian University Medical College, 30-663 Krakow, Poland; 2 Department of Pediatric Oncology and Hematology, University Children Hospital of Krakow, 30-683 Krakow, Poland; 3 Department of Clinical Immunology, Institute of Pediatrics, Jagiellonian University Medical College, 30-663 Krakow, Poland; 4 Department of Medical Genetics, Faculty of Medicine, Jagiellonian University Medical College, 30-663 Krakow, Poland; 5 Department of Epidemiology and Preventive Medicine, Faculty of Medicine, Jagiellonian University Medical College, 31-034 Kraków, Poland; 6 Department of Pediatric Oncology, Hematology and Transplantology, Poznan University of Medical Sciences, 60-572 Poznan, Poland; 7 Department of Pediatric Oncohematology, Clinical Province Hospital of Rzeszow, 35-301 Rzeszow, Poland; 8 Department of Pediatrics, Institute of Medical Sciences, Medical College, University of Rzeszow, 35-310 Rzeszow, Poland; 9 Department of Pediatrics and Hemato-Oncology, Pomeranian Medical University in Szczecin, 71-252 Szczecin, Poland; 10 Clinical Department of Pediatric Bone Marrow Transplantation, Oncology and Hematology, Wroclaw Medical University, 213 Borowska St., 50-556 Wroclaw, Poland; 11 Department of Pediatrics, Hematology and Oncology, Medical University of Gdansk, 80-210 Gdansk, Poland; 12 Departament and Clinic of Oncology, Pediatric Hematology, Clinical Transplantology and Pediatrics, Medical University of Warsaw, 02-091 Warsaw, Poland; 13 Departament of Pediatrics, Oncology and Hematology, Medical University of Bialystok, Kilinskiego 1, 15-089 Bialystok, Poland; 14 Department of Pediatric Hematology, Oncology and Transplantology, Medical University of Lublin, 20-090 Lublin, Poland; 15 Independent Laboratory of Genetic Diagnostic, Medical University of Lublin, 20-059 Lublin, Poland; 16 Department of Pediatrics, Oncology and Hematology, Medical University of Lodz, 91-738 Lodz, Poland; 17 Department of Pediatrics and Hematology and Oncology, Province Children’s Hospital, 10-561 Olsztyn, Poland; 18 Department of Pediatric Hematology and Oncology, Immunology and Transplantology, Collegium Medicum, Nicolaus Copernicus University Torun, 85-094 Bydgoszcz, Polandjstyczynski@cm.umk.pl (J.S.); 19 Department of Pediatrics, Medical University of Silesia, Upper Silesia Children’s Care Health Centre, 40-752 Katowice, Poland

**Keywords:** myeloid leukemia in Down syndrome, children, outcomes, prognostic factors

## Abstract

**Background:** Significant progress has been achieved in the treatment of children with myeloid leukemia associated with Down syndrome (ML-DS). We aimed to evaluate treatment outcomes and prognostic factors in children with ML-DS treated in Poland. **Methods:** Thirty-six children with ML-DS treated at pediatric hematology and oncology centers in Poland according to the ML-DS 2006 protocol based on the AML-BFM backbone between 2015 and 2024 were retrospectively analyzed. **Results:** The estimated 5-year event-free survival (EFS) and overall survival (OS) were 84.3% (95%CI: 72.5–98.1%) and 87.9% (77.4–99.8%), respectively. Although children with a complex karyotype showed numerically lower survival estimates than those with isolated constitutional trisomy 21 or additional cytogenetic abnormalities (OS: 60% [95%CI: 29.3–100%] vs. 90% [73.2–100%] vs. 92.3% [78.9–100%], *p* = 0.24; EFS: 60% [95%CI: 29.3–100%) vs. 75% [49.6–100%] vs. 92.3% [78.9–100%], *p* = 0.26), *p* = 0.26), these differences were not statistically significant. MRD positivity (≥0.1%) after the second induction cycle was identified as an adverse prognostic factor in the univariable analysis (HR = 31.1, 95%CI: 2.74–353.2; *p* = 0.006). **Conclusions:** Persistent MRD positivity after the second induction cycle may identify children with ML-DS at increased risk of treatment failure. Larger multicenter studies are needed to validate the prognostic significance of MRD and recurrent molecular alterations to optimize risk-adapted treatment strategies.

## 1. Introduction

Children with myeloid leukemia associated with Down syndrome (ML-DS) have a far better prognosis compared to children without Down syndrome (DS); however, when relapsed, the result of the treatment is extremely poor [1,2,3,4,5,6,7,8]. Historically, patients with Down syndrome had an increased incidence of early and late toxicity-related deaths. Increased sensitivity to therapy was frequently observed in this population [9,10]. Since the early 1990s, gradual reduction in therapy intensity in patients with ML-DS has led to significant improvement in treatment outcomes due to a reduced treatment failures and toxicity [9,10,11,12]. Many collaborative leukemia groups have achieved excellent treatment outcomes, with overall survival (OS) and event-free survival (EFS) rates of 87–95% and 83–95%, respectively [13,14,15,16,17,18]. Significant reduction in treatment-related mortality (TRM) from nearly 15–20% to 1–3% was also observed. This reduction was achieved primarily by reducing treatment intensity and introducing advanced supportive measures. However, severe infections and heart failure remain the leading causes of TRM in patients with Down syndrome [2,14,18].

Further reduction in treatment unfortunately carries the risk of treatment failure. In the Children’s Oncology Group (COG) AAML1531 trial, measurable residual disease (MRD), measured by multiparameter flow cytometry (FC) at the end of the first cycle of induction therapy, was used to stratify treatment intensity. MRD-negative patients (<0.05%) did not receive high-dose cytarabine, which ultimately led to decrease in EFS compared to a previous study (COG AAML043), with 2-year EFS 85.6% vs. 93.5% and 2-year OS 91% vs. 96.8%, respectively. Intensification of chemotherapy in patients with ML-DS and positive MRD not only did not improve survivals but also increased the incidence of treatment-related toxicities such as febrile neutropenia and sepsis [16].

As previously reported, in Poland, in the years 2005–2015, pediatric patients with ML-DS were treated according to the AML-BFM 2004 Interim protocol [18]. Compared to the therapy in children without DS, the AML-BFM 2004 Interim protocol included a reduction in anthracycline doses (cumulative dose of idarubicin from 19 to 13 mg/m^2^ and cumulative dose of mitoxantrone from 10 to 7 mg/m^2^). Despite the reduction in treatment intensity, the outcomes were comparable to those reported by other national study groups at that time, with a 5-year OS of 79 ± 7% and a 5-year EFS of 76 ± 7% [13,18,19]. However, treatment-related mortality remained high at 20%. In subsequent years, the ML-DS 2006 protocol, which incorporated further reductions in chemotherapy intensity, was introduced to reduce the substantial burden of treatment-related toxicity in patients with ML-DS and further improve treatment outcomes. The objective of the presented study was to analyze treatment outcomes and prognostic factors in children with ML-DS treated in Poland between 2015 and 2024.

## 2. Materials and Methods

We retrospectively analyzed the outcome of 36 children with ML-DS registered in the Polish Pediatric Leukemia and Lymphoma Study Group data base from 2015 to 2024 and treated according to the ML-DS 2006 therapeutic protocol. Therapy consisted of 4 cycles of multiagent chemotherapy blocks. Compared to the previously used AML-BFM 2004 Interim protocol, further deintensification of therapy was made. The cumulative dose of etoposide was reduced from 950 mg/m^2^ to 450 mg/m^2^. Furthermore, no maintenance therapy was administered, and the intrathecal cytarabine dose was reduced from 11 to 4 doses. Of the 36 patients included in the study, 35 (97.2%) received frontline treatment according to the ML-DS 2006 induction backbone. One patient (2.8%) with severe cardiac failure was considered ineligible for standard intensive induction chemotherapy and received an individualized regimen comprising venetoclax and azacitidine.

Therapy regimen and cumulative doses of cytostatic drugs are presented in Figure S1, Supplementary Materials.

In the study, commonly accepted definitions for conditions such as complete remission, primary refractory disease, relapse, early death, and treatment-related death were used. Complete remission was defined as less than 5% of blasts in bone marrow aspirate smears, an absolute neutrophil count > 1 × 10^9^/L, platelets 100 × 10^9^/L in peripheral blood, and no evidence of extramedullary involvement. Primary refractory leukemia was defined as failure to achieve complete remission after completion of the two induction cycles.

Relapse was defined as a recurrence of more than 5% unequivocal leukemic blasts in a representative bone marrow identified by microscopic and/or flow cytometry methods and/or evidence of leukemic infiltration of any site. Relapse occurring within 18 months after initial diagnosis was classified as an early relapse, while relapse after 18 months after diagnosis was defined as late. Early death was defined as death of any cause that occurred within 42 days after initial diagnosis. Death of the patient at the time of remission or absence of progression was defined as treatment-related mortality.

Cytogenetic analyses were performed in local laboratories, while molecular analysis of genetic alterations in leukemic blasts and mutation status in the *GATA1* gene were performed centrally in the Department of Medical Genetics, Institute of Pediatrics, Jagiellonian University medical College, Krakow, Poland. Between 2015 and the first half of 2021, *GATA1* analysis was performed using Sanger sequencing; in the second half of 2021, next-generation sequencing (NGS) was introduced. Nucleotide variants in exons 2, 3, and 4 of the *GATA1* gene were detected using a 3500 Genetic Analyzer (Applied Biosystems, Foster City, CA, USA). However, this method did not permit the reliable detection of large deletions or duplications, and its sensitivity was insufficient to identify somatic variants at low variant allele frequencies (VAFs), particularly those below approximately 10–20%. Therefore, to improve variant detection sensitivity, Sanger sequencing was replaced with an NGS-based approach using the Archer VARIANTPlex Myeloid Panel (ArcherDX, Boulder, CO, USA). According to the manufacturer’s specifications, this assay enables the detection of variants at VAFs of approximately 1–5%. Results of *GATA1* mutation status was available in 35 patients (97%).

The International System for Human Cytogenetic Nomenclature was used to describe cytogenetic assessment. A complex karyotype was recognized according to the ML-DS 2006 protocol and defined as the presence of at least three independent cytogenetic abnormalities, excluding constitutional trisomy 21, without favorable genetics and without *KMT2A* rearrangement.

Flow cytometry assessment of bone marrow samples was performed centrally in the Department of Clinical Immunology, Institute of Pediatrics, Jagiellonian University Medical College. Monitoring of MRD by multiparameter flow cytometry was performed before each chemotherapy cycle (assessment of response to the previous cycle) and included four assessment time points (days 28, 56, 84, and 112 from the start of therapy). A threshold of 0.1% or more of detected leukemic blasts relative to total nucleated cells was defined as MRD positivity. The 0.1% threshold was selected in accordance with the European ML-DS/AML-BFM approach and commonly applied MFC-MRD definitions in AML.

Centralized MFC-based MRD assessment results were obtained for 26 of the 36 patients (72.2%) after the first induction phase and for 29 of the 36 patients (80.5%) after the second induction phase. The absence of results was primarily due to the retrospective, multicenter nature of the study and the failure to collect or deliver samples to the reference laboratory at the designated time points, as well as, in some cases, an insufficient quantity or inadequate quality of material for reliable analysis. No data imputation was performed, and analyses included only those patients for whom evaluable MRD results were obtained at each time point.

For the exploratory analysis of potential prognostic factors, age, WBC count at diagnosis, and bone marrow blast percentage were categorized using cutoffs prespecified before the outcome analyses: age > 2 versus ≤2 years, WBC count > 10 versus ≤10 × 10^9^/L, and bone marrow blasts ≥ 20% versus <20%. These thresholds were selected on clinical grounds and were not derived by optimizing group separation within the present dataset.

Observation of the patients was carried out from the date of ML diagnosis until death, loss of follow-up, or 1 June 2026 (end of follow-up), whichever comes first. Median observation time was 58.1 months (range: 9.9–116 months).

The English language of the manuscript was checked using Writefull (ThinqLab Ltd., London, UK).

The study protocol has been carried out in accordance with The World Medical Association Ethics Code (Declaration of Helsinki) for experiments involving humans and approved by the Ethics Committee of Jagiellonian University (protocol code: 118.0043.1.237.2025, date of approval: 18 June 2025).

### Statistical Analysis

The main endpoints in the study were event-free survival (EFS) and overall survival (OS). EFS was defined as the time from the date of ML diagnosis to the first failure event (disease progression, relapse, or death). OS was defined as the time from the date of initial diagnosis to death from any cause. For survival analyses stratified by MRD status after the first and second cycles, the time-to-event interval was measured from the date of completion of the respective induction cycle to the date of the event or censoring. Survival probabilities were estimated using the Kaplan–Meier method and are presented with 95% confidence intervals (95%CIs). Differences between survival curves were assessed using the log-rank test. To identify risk factors associated with outcomes, univariable Firth-corrected Cox proportional hazards regression models were applied because of the small number of events observed during the study. The proportional hazards assumption was assessed using Schoenfeld residuals. Patients who had no event were censored on their last follow-up date. The significance level of 0.05 was used in all statistical tests. Statistical analyses were performed with SPSS version 29.0 and R statistical computing environment (R Foundation for Statistical Computing, Vienna, Austria) version 4.6.1.

## 3. Results

### 3.1. Patient’s Characteristics

Among 36 children there were 18 boys and 18 girls. Median age at diagnosis was 2 years (0.9–4.9 years). Median of WBC at diagnosis was 5.6 × 10^9^/L (2.6–39). In the majority of patients, 28 (77.7%), the leukocyte count was below 10 × 10^9^/L. None of the children presented hyperleukocytosis at the diagnosis. The median number of leukemic blasts in the bone marrow was 22% (7–40%). Most children had the M7 megakaryoblastic leukemia phenotype (34 patients, 94.4%), one child had the M2 phenotype, and one child had the M4 phenotype. The clinical condition of the patients was generally good, except for one child, who had symptoms of severe heart failure due to congenital heart disease at the time of ML diagnosis. This child started therapy with a low dose of ARAC. In one child, at the time of diagnosis, infiltration of the central nervous system was observed; no other symptoms of extramedullary myeloid leukemia were reported in the remaining patients. Only five children (13.8%) had a confirmed transient abnormal myelopoiesis (TAM) preceding the onset of ML-DS in history, without a need for therapy implementation.

Karyotype analysis showed isolated constitutional trisomy 21 in 15 of 33 children. Eighteen children had leukemic blasts with acquired clonal abnormalities in addition to constitutional trisomy 21. In eight patients (24%) whole chromosome gain (chromosomes 7, 8 or 22) was detected. Complex karyotype and trisomy 8 were the most common cytogenetic abnormalities (in five and six children, respectively). All but one patient were examined for *GATA1* mutation, and most of the patients had mutated *GATA1* blasts (26; 72.2% vs. 9; 25%). In individual patients, the presence of *CDKN2A*, *SBDS*, *CTC1*, and *KMT2A::MLLT11*, *DEK::NUP214*, and *FLT3::ITD* genetic alterations as well as *WT1* mutations was detected at diagnosis. The relevant clinical and genetic data of the patients are shown in Table 1.

### 3.2. Outcomes of Therapy

All study patients achieved complete remission by the end of the second induction block. The estimated 5-year EFS and OS were 84.3% (95%CI: 72.5–98.1%) and 87.9% (95%CI: 77.4–99.8%), respectively (Figure S2A,B; Supplementary Materials). No early deaths were reported. In total, 5 of the 36 patients (13.8%) experienced relapse. All relapses were classified as early and were confined to the bone marrow. Most patients who relapsed were older than 2 years (4 of 5). All but one relapse occurred within the first year after diagnosis, with a median time to relapse of 7.9 months (range, 6.8–16.6 months). Four of the five children who relapsed died, primarily due to disease progression. The clinical, genetic, and treatment characteristics of the patients who relapsed are summarized in Table 2.

### 3.3. Genetic Alterations

**Figure 1:**
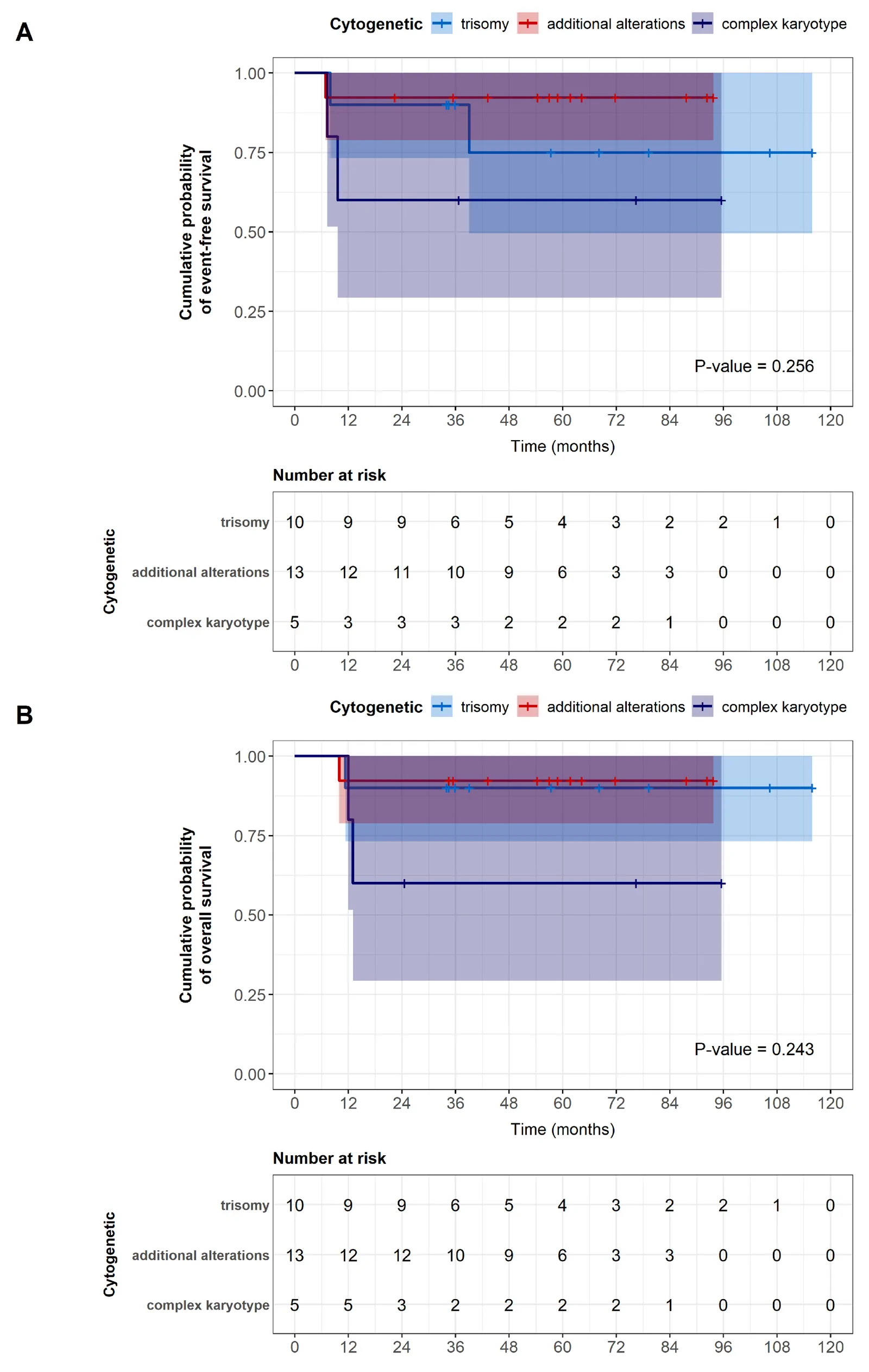
(**A**,**B**) Probability of event-free (EFS) and overall survival (OS) in children with isolated trisomy 21, additional cytogenetic alterations and complex karyotype.

Of the five children who relapsed, two had isolated constitutional trisomy 21, whereas three had additional cytogenetic abnormalities in leukemic blasts. Two patients had a complex karyotype. One child, a 2.3-year-old boy who relapsed 7.9 months after the initial diagnosis, acquired a complex karyotype at relapse, whereas no additional cytogenetic abnormalities had been detected at baseline. In addition, a *TP53* deletion was identified by FISH analysis at relapse in this patient. All patients who relapsed harbored *GATA1* mutations. In one patient, a *CDKN2A* deletion and a *JAK2* mutation were detected at relapse, whereas another patient harbored *CTC1* and *SBDS* mutations (Table 2). Children with a complex karyotype showed numerically lower survival estimates than those with isolated constitutional trisomy 21 or additional cytogenetic abnormalities, although the differences were not statistically significant: OS: 60% (95%CI: 29.3–100%) vs. 90% (73.2–100%) vs. 92.3 (78.9–100%), *p* = 0.24; EFS: 60% (29.3–100%) vs. 75% (49.6–100%) vs. 92.3% (78.9–100%, *p* = 0.26) (Figure 1A,B). However, only five patients had a complex karyotype, resulting in insufficient statistical power and a substantial risk of Type II error. Therefore, the absence of statistical significance should not be interpreted as evidence of no association.

### 3.4. MRD Analysis

**Figure 2:**
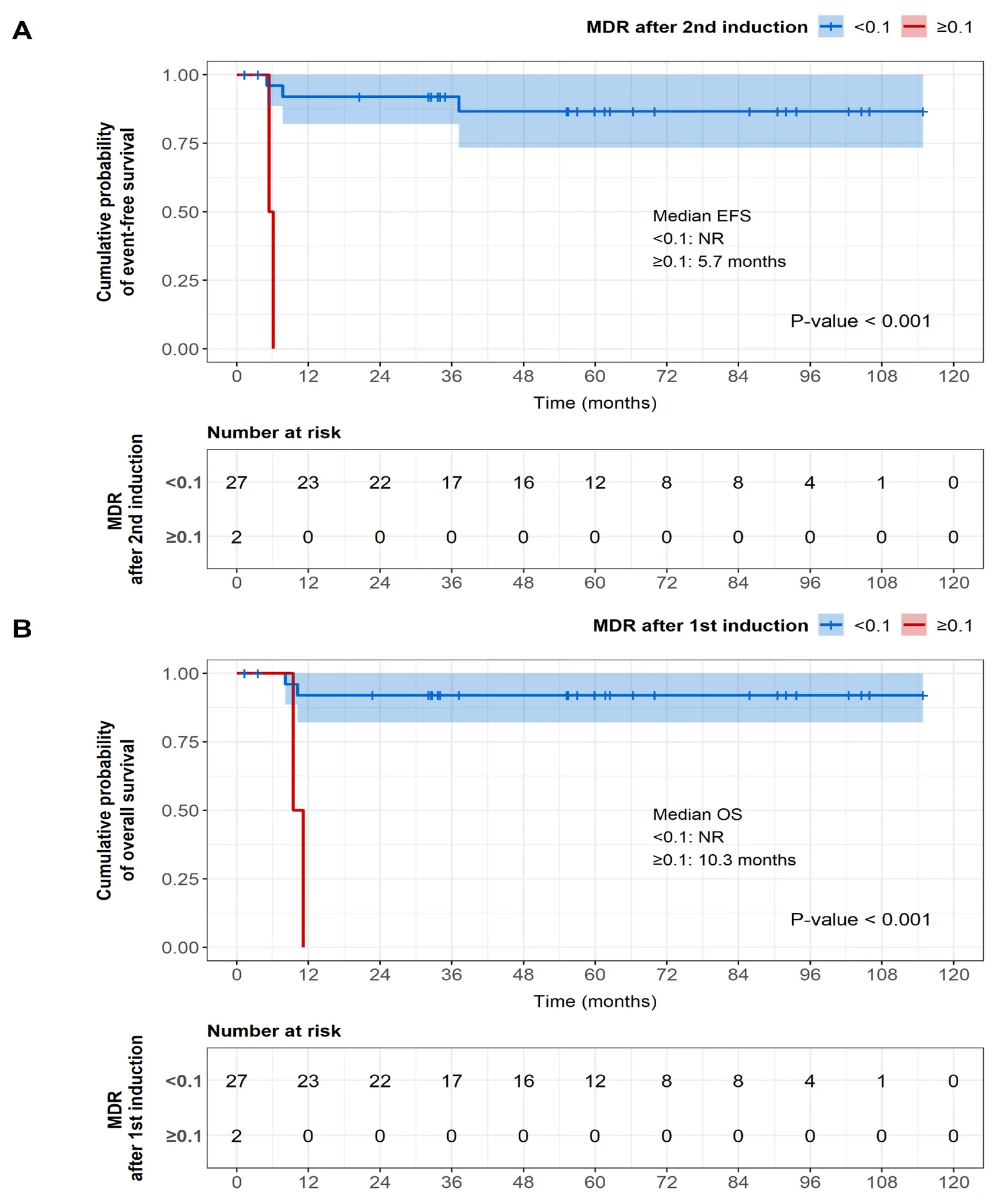
(**A**,**B**) Probability of event-free (EFS) and overall survival (OS) depending on MRD after the second induction.

In the present study, MRD was assessed after the first induction cycle (day 28) in 26 children (72%) and after the second induction cycle (day 56) in 29 children (80.5%). Patients with negative MRD (<0.1% blasts) after the second induction had significantly better outcomes than those with positive MRD (*p* < 0.001) (all patients with positive MRD reached outcomes before 5 years) and, for patients with negative MRD 5-year EFS, was 86.6% (95%CI: 73.4–100%), while 5-year OS was 86.6% (95%CI: 73.4–100%), as shown in Figure 2A,B.

After the first induction, patients with negative MRD had numerically higher 5-year EFS and OS estimates than MRD-positive patients; however, these differences were not statistically significant (5-year EFS: 80.2% [95%CI: 64.3–100%), *p* = 0.19; 5-year OS: 80.2% {95%CI: 64.3–100%], *p* = 0.14), as shown in Figure S3A,B (Supplementary Materials).

### 3.5. Prognostic Factors

Several potential predictors of event occurrence were evaluated using univariable Firth penalized Cox regression (Table S1, Supplementary Materials). MRD positivity (≥0.1%) after the second induction was the only factor significantly associated with an increased risk of an event (HR = 16.96, 95%CI: 2.55–112.79; *p* = 0.006). However, the wide confidence interval indicates considerable uncertainty in the magnitude of this association. No statistically significant associations were observed for age, sex, initial white blood cell count, bone marrow blast percentage, *GATA1* mutation status, additional cytogenetic abnormalities, complex karyotype, or MRD status after the first induction. Notably, the hazard ratio for *GATA1* mutation status remained highly imprecise despite the use of Firth’s penalization, as reflected by its particularly wide confidence interval, and should therefore be interpreted with caution.

## 4. Discussion

Less intensive chemotherapy regimens have markedly improved the prognosis of children with ML-DS, although treatment failure still occurs in approximately 10–15% of patients [13,14,15,16,17,18,19,20]. In this nationwide retrospective study, we evaluated the outcomes of 36 children treated predominantly according to the ML-DS 2006 treatment backbone in Poland between 2015 and 2024. Compared with our historical cohort, further reduction in treatment intensity was associated with improved survival (5-year EFS: 84.3% vs. 74.8%; 5-year OS: 87.9% vs. 79.2%). Notably, no treatment-related deaths were observed in this cohort (TRM, 0%; 0/36), despite the increased vulnerability of children with Down syndrome to treatment-related toxicity. However, given the limited sample size, this finding should be interpreted cautiously and should not be considered evidence that the treatment regimen carries no risk of treatment-related mortality.

Our results are consistent with those of the international ML-DS 2006 trial conducted by the AML-BFM, NOPHO, and DCOG study groups, in which the reported 5-year EFS and OS were 87% and 89%, respectively. Although the relapse rate in our cohort was slightly higher than in the ML-DS 2006 study (13.8% vs. 5.3%), this difference should be interpreted cautiously because of the limited sample size. Importantly, most of the relapses (four out of five) occurred within the first year after diagnosis and four of the five affected children died because of disease progression, suggesting that relapse remains the principal cause of treatment failure in ML-DS despite improvement of overall survival.

The ML-DS 2006 study demonstrated that poor early treatment response (>5% bone marrow blasts after the first induction cycle) and gain of chromosome 8 independently predicted treatment failure, whereas COG studies identified complex cytogenetics as an adverse prognostic factor [14,16]. In our cohort, MRD positivity assessed by flow cytometry after the second induction cycle emerged as the factor most strongly associated with adverse outcome, whereas MRD after the first induction cycle and cytogenetic abnormalities were not significantly associated with survival.

The optimal method, threshold and timing of MRD assessment in ML-DS remain unresolved. While the COG AAML1531 trial did not support MRD-MFC after the first induction cycle for treatment stratification, the interim analysis of the ongoing ML-DS 2018 study demonstrated that MRD assessed by next-generation sequencing (MRD-NGS) provides superior prognostic discrimination compared with MRD-FC [16,21]. In contrast, our results suggest that MRD positivity assessed by flow cytometry after the second induction cycle may still identify children at particularly high risk of relapse. Although only two patients remained MRD-positive after the second induction, both experienced early relapses, supporting the potential clinical relevance of this time point.

The optimal MFC-MRD threshold in ML-DS remains uncertain and has varied across cooperative studies. In the present study, MRD positivity was defined as ≥0.1%, in accordance with the European ML-DS/AML-BFM approach, whereas lower thresholds were used in COG studies, including >0.01% after induction I in AAML0431 and 0.05% for risk stratification in AAML1531 [13,16]. Therefore, patients with detectable MRD below 0.1% may have been classified as MRD-negative in our analysis, potentially overlooking prognostically relevant low-level disease. This difference should be considered when interpreting our findings and comparing them with results from other ML-DS cohorts. The differences between the study groups further highlight the lack of a universally validated MFC-MRD cutoff specifically established for ML-DS.

Although MRD positivity after the second induction was associated with inferior outcomes, this finding was based on a very small number of MRD-positive patients and was accompanied by considerable statistical uncertainty. It should therefore be interpreted as exploratory rather than conclusive.

In addition to treatment response, molecular profiling is becoming increasingly important for risk stratification in ML-DS. The JPLSG first identified recurrent alterations involving *CDKN2A*, *ZBTB7A*, *JAK2*, and *TP53*, which were subsequently validated by the Children’s Oncology Group as markers associated with relapse and poor survival, particularly when combined with MRD positivity [16,22,23]. We identified several of these adverse molecular abnormalities in our cohort, including *CDKN2A*/*JAK2* alterations in one child with early relapse and detection of a *TP53* deletion at relapse that had not been identified at diagnosis in another patient, suggesting possible clonal evolution during treatment. We also detected somatic *CTC1* and *SBDS* mutations in one relapsed patient. Although the prognostic significance of these alterations in ML-DS remains unknown, these findings emphasize the value of comprehensive molecular profiling, particularly at relapse, to improve risk stratification and better understand the biology of treatment resistance [24,25,26,27].

Of note, somatic *GATA1* mutations, which represent a molecular hallmark of ML-DS, were not detected in 9 of 35 tested patients (25.7%), a proportion higher than expected in molecularly confirmed ML-DS. The methodology used for *GATA1* testing changed during the study period. Initially, testing was performed using Sanger sequencing, whereas an NGS-based method was subsequently introduced. The limited analytical sensitivity of Sanger sequencing, particularly for variants with variant allele frequencies below approximately 10–20%, may have contributed to the relatively high proportion of patients without a detected *GATA1* mutation. Accordingly, the absence of a detectable mutation should not be interpreted as definitive evidence of *GATA1*-wild-type disease.

The univariable estimate for *GATA1* mutation status remained subject to considerable uncertainty despite the use of Firth’s penalized regression. The wide confidence interval indicates residual imprecision related to sparse data and the limited number of outcome events; therefore, no definitive conclusions regarding the prognostic significance of *GATA1* mutation status can be drawn from this analysis.

The present study has several limitations. First, centralized MRD-MFC results were not available for all patients, particularly after the first induction cycle. Given the small cohort, missing data at clinically relevant timepoints may have introduced selection bias, especially because the missingness could not be assumed to be random. Therefore, the prognostic associations identified in the evaluable subgroup should be interpreted cautiously and require validation in larger prospective cohorts with complete and standardized MRD assessment.

Second, although most patients were treated according to the ML-DS 2006 backbone, one patient received frontline venetoclax plus azacitidine because severe cardiac failure precluded standard intensive chemotherapy. This deviation introduced a limited degree of treatment heterogeneity. However, because it involved only one patient, a formal sensitivity analysis excluding this case would have had limited statistical value given the small cohort and low number of outcome events. Furthermore, this patient’s outcome may have been influenced by both the alternative treatment and the underlying cardiac comorbidity.

Third, the analysis of cytogenetic risk factors was exploratory and substantially constrained by the small subgroup sizes, particularly the complex-karyotype group, which included only five patients. Consequently, the study lacked sufficient statistical power to reliably detect associations between cytogenetic abnormalities and survival, and the non-significant findings may represent Type II errors. Thus, no definitive conclusions regarding the prognostic significance of complex karyotype or other cytogenetic abnormalities can be drawn from this cohort.

Finally, the number of observed events was limited; therefore, penalized Cox proportional hazards model using Firth’s correction was used to reduce small-sample bias and to address potential separation issues resulting from the limited number of events.

## 5. Conclusions

In conclusion, this nationwide cohort demonstrates that favorable survival outcomes can be achieved in children with ML-DS treated predominantly with reduced-intensity chemotherapy, with no treatment-related deaths observed. Persistent MRD positivity after the second induction was strongly associated with treatment failure; however, this finding was based on only two MRD-positive patients and should be regarded as hypothesis-generating. Larger prospective studies incorporating standardized MRD assessment, clearly defined analytical thresholds, and comprehensive genomic profiling are needed to validate risk factors and identify patients who may benefit from treatment modification.

## Figures and Tables

**Table 1:** Clinical and genetic characteristics of the patients (*n* = 36).

Feature	All Patients
**Gender**	
Female Male	18 (50%) 18 (50%)
**Median age at diagnosis—years (range)**	2 (0.9–4.9)
≤2 years >2 years	17 (47.2%) 19 (52.8%)
**FAB classification**	
M2 M4 M7	1 1 34
**TAM history**	
Yes No No data	5 30 1
**WBC at diagnosis (×10^9^/L)** median (range)	5.6 (2.6–39)
**WBC ≤ 10 × 10^9^/L**	28
**WBC > 10 × 10^9^/L**	7
**Platelets at diagnosis (×10^9^/L)** median (range)	38 (5–499)
**Blasts in peripheral blood at diagnosis (×10^9^/L)** median (range)	0.66 (0–79)
**Blasts in bone marrow at diagnosis (%)** median (range)	23 (7–40)
**Genetic abnormalities**	*n* = 33
Trisomy 21 only Trisomy 22 Trisomy 8 Trisomy 7 Complex karyotype Other No data	15 (45.5%) 1 (3.0%) 6 (18%) 1 (3.0%) 5 (15%) 5 (15%) 3
**Molecular genetics**	
*KMT2A::MLLT11* *DEK::NUP214* *FLT3-ITD* *CDKN2A* *WT1* *SBDS* *CTC1*	1 1 1 1 1 1 1
***GATA1* mutations**	
Yes No No data	26 (72.2%) 9 (25%) 1 (2.8%)
**Congenital heart defect**	
Yes No No data	4 29 3

**Table 2:** Characteristics of the patients with relapse ML-DS.

	Sex	Age	FAB	Genetic Alterations	WBC (×10^9^/L)	Therapy	Special Information	MRD-FC After 1 Induction (28 Day)	MRD-FC After 2 Induction (56 Day)	Time of Relapse After Diagnosis (Months)	Type of Relapse	Genetic Alterations at Relapse	Treatment of Relapse	Outcome
1	M	2.9 y	M7	47,XY,del(11)(q23),+21c [2] /49,XY,+8,del(11)(q23),+15,+21c [6]/ 47,XY,+21c [15] *GATA1* mutation	16.4	ML-DS2006		<0.1%	<0.1%	9.5	BM	*GATA1* mutation	FLA	Dead PD
2	M	2.1 y	M7	47,XY,16,+21c,+mar(6)/47,XY,+21c(5) *GATA1* mutation	2.58	ML-DS2006	TAM in history	<0.1%	<0.1%	6.8	BM	*GATA1* mutation *SBDS* and *CTC1* mutation	VenAza	Dead PD
3	F	1.8 y	M7	47, XX, der(9)(p12)t(9:11)(q34;q13), add(15)(p11.2), ins(?;20)(?;q12q13.?2),+21c [14]/47,idem, der(10)t(10;11)(q26;q13){6]/47, idem, der (21)t(11;21)(q13;p11.2) [3]/47,XX,+21c [4] *GATA1* mutation	4.4	ML-DS2006		<0.1%	**0.4%**	7.2	BM	*GATA1* mutation *CDKN2A* and JAK2 mutation	IDA-FLA, FLA + GO, GO	Dead PD
4	M	2.3 y	M7	47 XY + 21 (20) *GATA1* mutation	3.53	ML-DS2006		**0.2%**	**0.1%**	7.9	BM	47,XY,+21,add(9)(p?), t(10;19)(?;?), der(11)t(11;14;21)(p?;?;p?), der(21)t(11;14;21)(p?;?;p?), del(17)(p13) *GATA1* mutation *TP53* mutation	AZA + GO, IDA-FLA, TVTC + Ven	Dead PD
5	F	1.1 y	M7	47 XX + 21 (18) *GATA1* mutation	13	VenAza/ML-DS2006	Cardiac failure	<0.1%	<0.1%	16	BM	*GATA1* mutation	VenAza, alloHSCT	Alive in 2. CCR

M—male, F—female, y—years, TAM—transient abnormal myelopoiesis, BM—bone marrow, VenAza—Venetoklaks and Azacitidine, Ven—Venetoklaks, IDA-FLA—Idarubicine, Fludarabine, GO—gemtuzumab ozogamycin, TVTC—Clofarabine, Topotecan, Vinorelbine, and Thiotepa, alloHSCT—allogenic hematopoietic stem cell transplantation, PD—progression disease, ML-DS 2006—therapy protocol.

## Data Availability

The data that support the results of this study are available from the corresponding author upon reasonable request.

## Supplementary Materials

The following supporting information can be downloaded at: https://www.mdpi.com/article/10.3390/jcm15197548/s1. Figure S1. Therapy regimens according to the ML-DS 2006 Protocol. Figure S2. Probability of event-free (EFS) and overall survival (OS) for ML-DS patients. Figure S3. Probability of event-free (EFS) and overall survival (OS) depending on MRD after the first induction. Table S1. Penalized Firth regression analysis and clinical and genetic factors for the event incidence.
