# Supplementary material for: Treatment Outcomes and Prognostic Factors in Children with Myeloid Leukemia Associated with Down Syndrome: A Nationwide Polish Retrospective Study
Source: J Clin Med. 2026 Sep 28;15(19):7548. doi: 10.3390/jcm15197548 (PMC13665886; doi:10.3390/jcm15197548)
Supplement: Supplementary file 1 [file jcm-15-07548-s001.zip › jcm-4511275-supplementary.pdf]

Figure S1. Therapy regimens according to the ML-DS 2006 Protocol

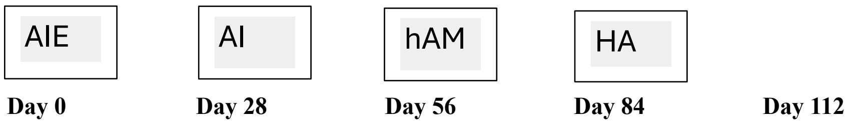

Induction phases:

AIE: cytarabine 100 mg/m2/day continuous infusion on days 1 and 2, followed by 30 min infusion every 12 h on days 3–8; idarubicin 8 mg/m2/day, 30 min infusion days 3, 4, and 5; etoposide 150 mg/m2/day, 60 min infusion on days 6–8. **AI:** cytarabine 500 mg/m2/day, 96-hours infusion on days 1–4; idarubicin 5 mg/m2/day, 30 min infusion on days 3 and 5.

Consolidation phases: haM: cytarabine 1 g/m2/dose, 3 h infusion every 12 h on days 1–3 (6 doses); mitoxantrone 7 mg/m2/day, 30 min infusion on days 3 and 4. **HA**: cytarabine 3 g/m2/dose, 3 h infusion every 12 h on days 1–3 (6 doses);

**Cumulative doses**	**mg/m^2**
Cytarabine	27 400
Etoposide	450
Idarubicin	34
Mitoxantrone	14

Chemotherapy for children younger than 12 months of age, or weighing 10 kg, was calculated based on body weight.

Intrathecal cytarabine was administered at the start of each treatment block (4 doses in total, 20-40 mg per dose adapted to age) as a CNS prophylaxis.

Figure S2. Probability of event-free (EFS) and overall survival (OS) for ML-DS patients

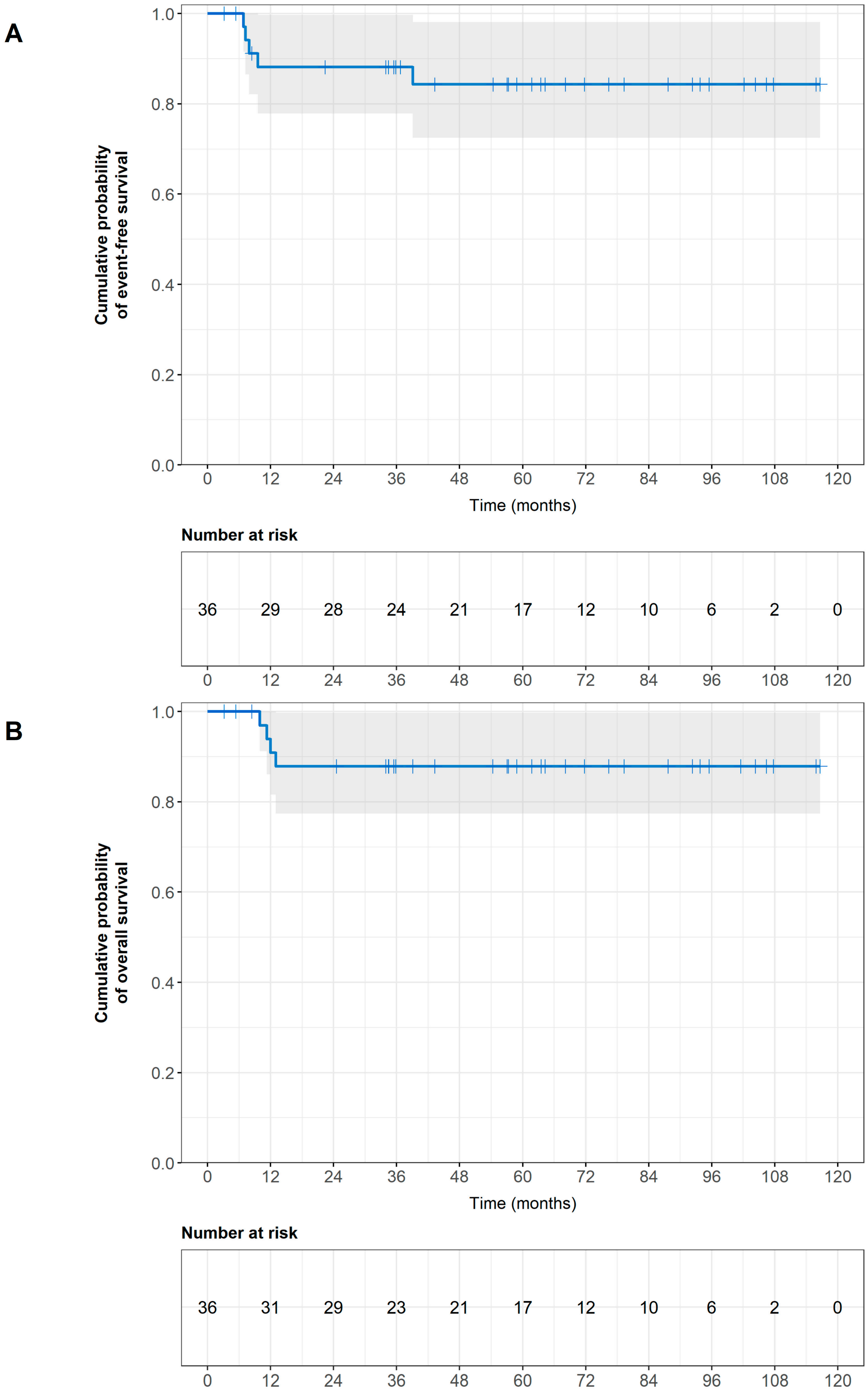

Figure S3. Probability of event-free (EFS) and overall survival (OS) depending on MRD after the first induction.

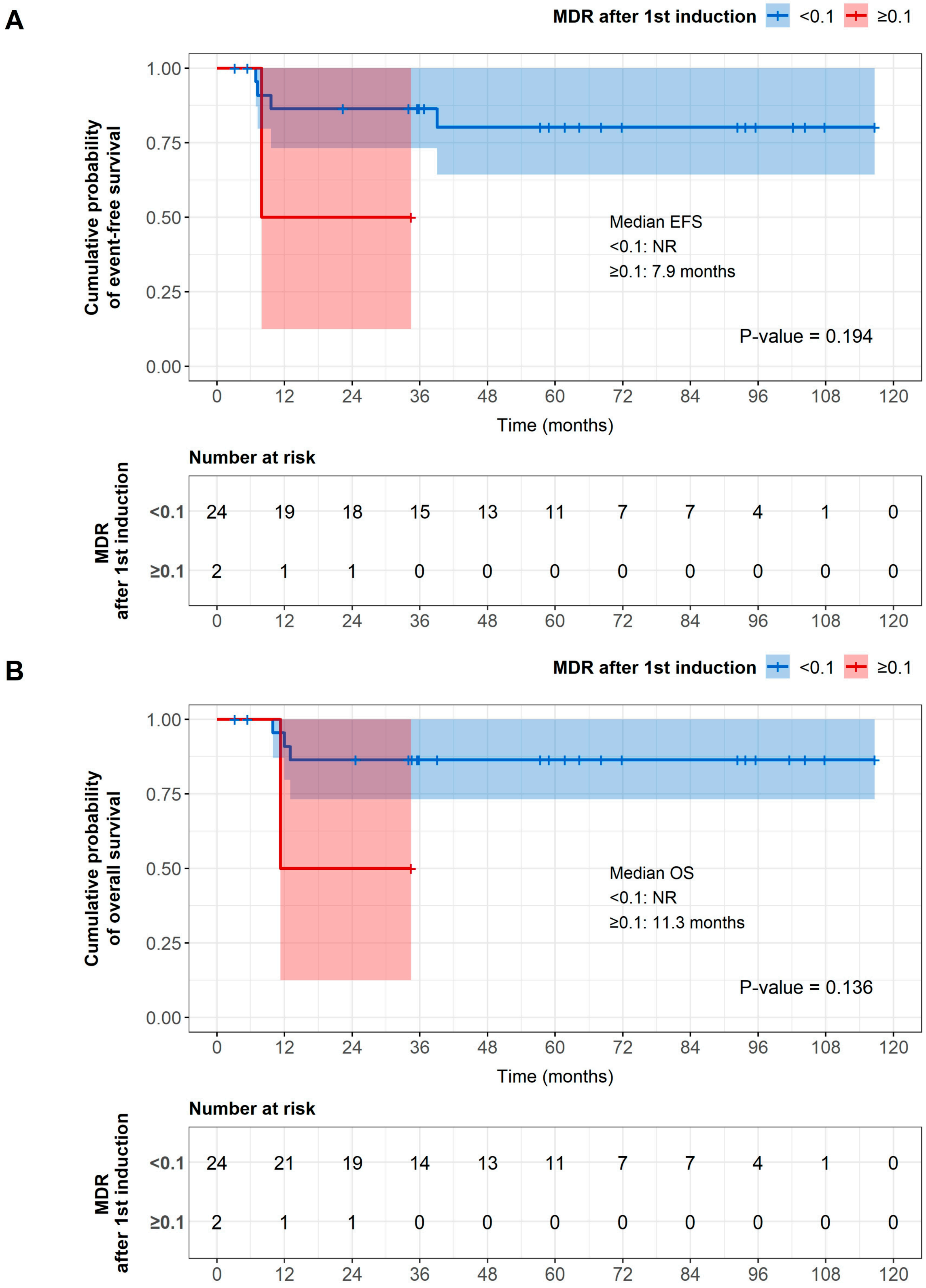

Table S1. Penalized Firth regression analysis and clinical and genetic factors for the event incidence

Univariable analysis			
**n= 36**	HR	95%CI	P
**Sex (M vs F)**	0.36	0.04-2.22	0.28
**Age (> 2y vs ≤ 2 y)**	2.13	0.35-21.98	0.42
**WBC (≤ $10x10^9$/l vs > $10x10^9$/l)***	1.51	0.15-9.18	0.68
**BM blasts (≥20% vs <20%)***	2.18	0.36-22.54	0.40
**GATA1 (pos vs neg)***	3.78	0.40-500.77	0.29
**Trisomy 21 ***	1.0 (ref.)		-
**Additional abnormalities**	0.77	0.06-9.54	0.83
**Complex karyotype**	3.34	0.44-36.57	0.23
**MRD after 1st induction* (≥0.1% vs <0.1%)**	6.17	0.59-38.71	0.11
**MRD after 2nd induction* (≥0.1% vs <0.1%)**	16.96	2.55-112.79	**0.006**

HR-Hazard ratio, CI – confidence interval, M-male, F-female, WBC- white blood count, BM- bone marrow, y-year, neg- negative, pos-positive, MRD – measurable residual disease by flow cytometry

* Missing data: n=1 for WBC and GATA1; n=2 for BM blasts; n=8 for Cytogenetic; n=10 for MRD after 1st induction and n=7 for MRD after 2nd induction.
